# Variations of Habitat Quality and Ecological Risk and Their Correlations with Landscape Metrics in a Robust Human Disturbed Coastal Region—Case Study: Xinggang Town in Southern China

**DOI:** 10.3390/ijerph20042837

**Published:** 2023-02-06

**Authors:** Huiqing Han, Zhihua Su, Guangbin Yang

**Affiliations:** 1 School of Architecture and Urban Planning, Guizhou Institute of Technology, Guiyang 550003, China; 2 School of Management Science, Guizhou University of Finance and Economics, Guiyang 550025, China; 3 School of Geographical and Environmental Sciences, Guizhou Normal University, Guiyang 550025, China

**Keywords:** habitat quality, ecological risk, landscape metrics, spatial–temporal change, distance gradient, coastal region, Xinggang town

## Abstract

This paper explores how landscape risk and habitat quality vary in coastal areas with strong anthropogenic disturbance based on a case study. We analyze the temporal–spatial variations of habitat quality and ecological risk in the coastal region by adopting the methods of theInVEST model and the ecological risk index. The correlations of habitat quality and ecological risk with landscape metrics are subsequently quantified. The results indicated the presence of obvious distance gradients in relation to the deterioration of habitat quality and the increase in ecological risk. Moreover, the gradient area close to the coastline exhibits significant habitat quality and ecological risk changes. The majority of landscape metrics show positive correlations with habitat quality and ecological risk, and these correlations vary with the distance gradients. Since the rapid urbanization of the coastal region, the marked expansion of built-up land and decrease in natural landscapes has significantly impacted the landscape pattern index and, consequently, changed the habitat quality and ecological risk level.

## 1. Introduction

Over the past few decades, the coastal landscape pattern has significantly altered due to the adverse effects of human activities. As a result, the material and energy flows of the ecosystem have been severely disturbed and destroyed [1,2]. Landscape fragmentation and complex landscape structures attributed to extensive human disturbance will reduce habitat quality and increase ecological risk. This is a vital problem facing coastal regions with robust human activities [3,4]. The analysis of habitat quality and ecological risk are critical to boosting the sustainable development of the social economy, the rational use of land resources and ecological planning in coastal regions [5,6,7].

There have been a number of studies conducted globally on coastal habitat quality and ecological risk. Habitat quality and ecological risks have been assessed in different coastal regions worldwide [8,9,10,11]. The assessment of habitat quality primarily focuses on single species [12] and biological communities [13]. The methods of field sampling, landscape ecology and ecological model have been extensively employed for habitat quality assessment. For example, the habitat quality of juvenile halibut has been assessed in the southeastern Bering Sea based on the trawl and benthic sampling approaches [14]. Two different index systems are adopted to assess the quality of benthic habitats in the adjacent waters of Xiaoqing River in Laizhou Bay [15]. The Integrated Valuation of Ecosystem Services and Tradeoffs (InVEST) model was employed to assess the habitat quality in the coastal regions of the Yellow River Delta in China [11]. The InVEST model was developed by Stanford University, the World Wide Fund for Nature and the Nature Conservancy for the assessment of multiple ecosystem services [16]. The model has the advantages of less parameter requirements, easy access to parameters and simple data processing. Compared to other models, InVEST allows for the spatial representation of the habitat quality assessment results with the help of easily available land use data [17]. As a result, the model has been widely used in habitat quality assessments around the world [18,19]. The research scope of ecological risk assessments has extended from micro assessments at the population and community levels to macro assessments at the landscape and regional levels [20,21,22]. The assessment methods of ecological risk consist of the multi-index and landscape ecology methods. For example, Hua [23] employed landscape metrics to assess the landscape ecological risk of Xiamen in coastal China. Li [24] assessed the ecological risk of heavy metals in soils of Hangzhou Bay, China, using the potential ecological risk index.

The fields of habitat quality and ecological risk have aroused the attention of global scholars. As indicated by existing studies, the quality of plankton habitat in urban rivers has been reduced with the development of coastal cities [5]. The increase in cultivated, aquaculture and industrial land, and the decrease in wetland areas and unused land has resulted in the significant decline of habitat quality in coastal regions [4]. Beach restoration has been reported to improve the habitat quality of horseshoe crabs and shorebirds [25]. Moreover, previous research has demonstrated the impacts of river diversion on oyster habitat quality in the coastal regions of the Mississippi River Basin [26]. The increase in built-up areas and farmland and the decrease in natural grassland and forest has increased the level of ecological degradation [27]. Furthermore, the expansion of urbanization in coastal regions has enhanced the area of high ecological risk regions [28]. Current research has confirmed that landscape patterns have serious impacts on coastal ecosystems. However, the correlations of habitat quality and ecological risk with landscape metrics remain unclear. In addition, the distance gradient characteristics of variations in habitat quality and landscape risk in coastal areas have yet to be reported. Therefore, we address the aforementioned gaps in the current literature by presenting a case study. The contributions of this study include (1) revealing the distance gradient characteristics of habitat quality and ecological risk variations from a temporal–spatial perspective, and (2) quantifying the correlations of habitat quality and ecological risk with landscape metrics based on the Pearson and spatial correlations methods.

The Chinese coastline is one of the longest worldwide. China’s coastal regions are populated areas and key economic development zones due to their location advantage, resource endowment, topography and history. Coastal regions only take up 14% of China’s total land area, whereas they contain over 42% of the country’s population and more than 60% of total gross domestic product [29]. Over the past few years, rapid economic development and population growth have led to the deterioration of ecological conditions in China’s coastal regions, which has seriously threatened their sustainable development [30]. Although habitat quality and ecological risk have been assessed in the coastal regions of China [11,21], small-scale coastal regions strongly disturbed by human activity in China have rarely been studied. 

Accordingly, this paper takes Xinggang Town, Guangxi Province, South China, as a typical coastal region disturbed by robust human activities. The spatial–temporal variations of habitat quality and ecological risk and their correlations with landscape metrics in a robust human disturbed coastal region are analyzed. We propose the following hypotheses: (1) spatial–temporal variations of habitat quality and ecological risk in the coastal region are characterized by distance gradients (from coastline to inland); (2) differences are present in the spatial–temporal change characteristics between habitat quality and ecological risk in the coastal region; and (3) habitat quality and ecological risk are correlated with landscape metrics in coastal regions and the correlations vary with distance gradients. Hence, we evaluated habitat quality and ecological risk using the InVEST model and the landscape risk index, respectively. Thereby, the variations of habitat quality and ecological risk in different distance gradients are explored with the help of GIS technology. Finally, the Pearson and spatial correlations are employed to reveal the relationships of habitat quality and landscape ecological risk with landscape metrics.

## 2. Materials and Methods

### 2.1. Study Area

**Figure 1:**
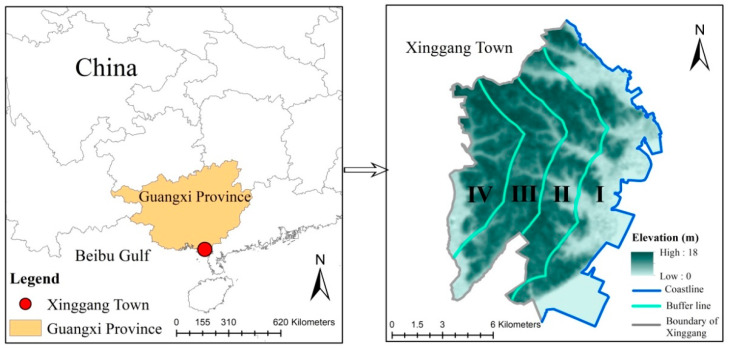
Location of the study area. Data source: derived from standard map provided by the Ministry of Natural Resources of China. (Gradient I (0–2 km), gradient II (2–4 km), gradient III (4–6 km) and gradient IV (>6 km) from the coastline to the inland).

Xinggang town (109°47′−109°59′ E, 21°48′−21°63′ N) is located in the south of Guangxi Province of South China, covering an area of 123.42 km^2^. This town is considered as a critical zone for the development in the Beibu Gulf of Guangxi Province due to its favorable conditions, including its natural harbor (31 km coastline), climate and topography, etc. (Figure 1). The altitude of this town ranges between 0 and 18 m, with a flat terrain. It has a subtropical monsoon climate area with an average annual temperature of 22.6 °C and an average annual rainfall of 1663.7 mm. In such an important agricultural production region, rice and vegetables are broadly cultivated as agricultural crops. Furthermore, there exist abundant shallow beach resources and diverse aquatic products. Xinggang Town has experienced rapid economic development and population growth in recent years, and the total population reached 65,000 by 2019.

### 2.2. Data Sources and Processing

**Figure 2:**
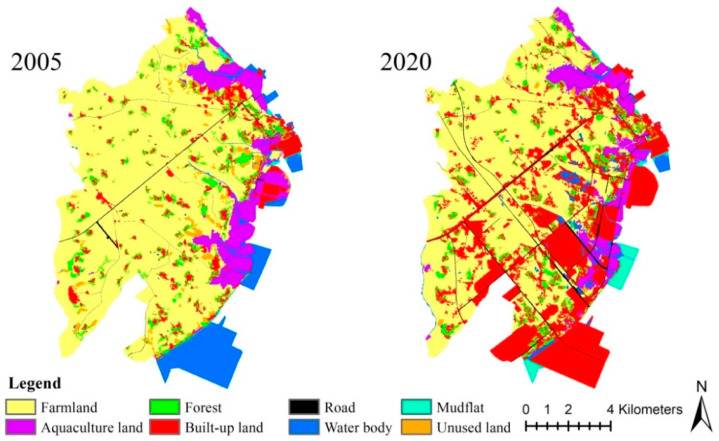
Spatial distribution of landscape types. Data source: remote sensing interpretation.

We employ remote sensing images (1 m spatial resolution) from the Pleiades satellite, collected in 2005 and 2020, and obtained from Europe Astrium. ERDAS (version X, ERDAS, Inc., Norcross, GA, USA) was employed for the geometric correction, image registration and image mosaicking based on a 1:10,000 topographic map and field surveys. Considering the characteristics exhibited by the study area, visual interpretation was used to classify the landscape types into the following eight types: farmland, aquaculture land, forest, built-up land, road, water body, mudflat and unused land. After the field corrections, the Kappa coefficient of landscape type data exceeded 0.9, demonstrating compliance with the data accuracy requirements of the study. Under the scope of the study area, Xinggang Town falls to gradient I (0–2 km), gradient II (2–4 km), gradient III (4–6 km) and gradient IV (>6 km) with an interval of 2 km from the coastline to the inland (Figure 1 and Figure 2).

### 2.3. Methods

#### 2.3.1. Habitat Quality Assessment

Landscape type data were adopted to assess regional habitat quality in accordance with the impact intensity, distance and sensitivity of different threat factors in the habitat quality module of InVEST [31,32]. In order to operate the module, the habitat type and the intensity and distance of the threat factor effect on the respective habitat are required, as well as the sensitivity of the respective habitat to the threat factors. The farmland, aquaculture land, built-up land and roads obtained from the remote sensing interpretation are considered as the landscape types that have severely been disturbed by humans, and they are denoted as the threat factors. The sensitivity of the threat factors to habitat, the distance and weight of the effect of the threat factors and other parameters are determined in accordance with the expert score, the recommended value of the model user’s guide [33] and related literature [34,35]. The parameter settings of the habitat quality module are listed in Table 1 and Table 2. The habitat quality and habitat degradation degree are expressed as Equations (1) and (2), respectively:

(1) $$Q_{xj}=H_{j}\left(1-(\frac{D_{xj}^{z}}{D_{xj}^{z}+k^{z}})\right)$$

(2) $$D_{xj}=\sum_{r=1}^{R}\sum_{y=1}^{Y}\left(\frac{w_{r}}{\sum_{r=1}^{R}w_{r}}\right)r_{y}i_{rxy}\beta_{x}S_{jr}$$

where *Q_xj_* denotes the habitat quality; *H_j_* expresses the habitat suitability index; *D_xj_* is the habitat degradation degree; *Z* is equal to 2.5; *k* is equated with half of the grid cell resolution; *W_r_* represents the weight of the threat factors; *ry* is the number of ecological threat factors; *i_rxy_* is the effect of threat factor r in grid y on grid *x*; *β_x_* is the degree of protection (not considered in this paper); and *S_jr_* is the sensitivity of the threat factors to different habitats. The assessment results range from zero to one: the closer to one, the higher the habitat quality; the closer to zero, the lower the habitat quality.

#### 2.3.2. Ecological Risk Assessment Methods

From the perspective of the landscape structure of the regional ecosystem, ecological risk at the landscape level is assessed with the area proportion of the respective landscape component, the landscape structure index and the landscape fragility index [36,37], as follows:

(3) $$ERI_{k}=\sum_{i=1}^{m}\frac{A_{ki}}{A_{k}}LL_{i}$$

(4) $$LL_{i}=U_{i}\times S_{i}$$

(5) $$U_{i}=aC_{i}+bF_{i}+cD_{i}$$

where *ERI_k_* is the ecological risk index of the *k* fishnet; *A_ki_* denotes all fishnet landscape areas; *A_k_* is the area of the *k* fishnet; m expresses the number of landscape types in the fishnet; *LL_i_* is the landscape ecological loss index; *U_i_* represents the landscape disturbance index; *C_i_* denotes the landscape fragmentation degree; *F_i_* is the landscape separation degree; *D_i_* is the landscape dominance degree; *a*, *b* and *c*, respectively, denote the weights of fragmentation degree, separation degree and dominance degree; *S_i_* is the vulnerability index of the landscape; and *a*, *b* and *c*, respectively, denote the weights of the fragmentation, separation and dominance degrees. Based on previous literature [38,39], 0.3, 0.2 and 0.5 are used as the values of *a*, *b* and *c*, respectively. Given the ability of the respective landscape type to resist external influences, vulnerability index was assigned as follows: water body = 7; mudflat = 6; aquaculture land = 5; farmland = 4; unused land = 3; forest = 2; built-up land; and road = 1. The settings of these parameters are mainly based on previous studies [40,41]. An excessively small fishnet size will greatly increase the statistical workload, and too large a fishnet size reduces the accuracy of the study results. Thus, taking into account the area of the study area, the calculation of ecological risk complies with the 400 m × 400 m fishnets in the study area. The fishnet tool in ArcGIS was adopted to generate 847 fishnets in the study area.

#### 2.3.3. Landscape Metrics Selection

We employ landscape metrics as the indicators to quantify the landscape pattern, as they can indicate the effect of human activities on landscape pattern [42]. We adopt six landscape metrics at the landscape level, namely, the number of patches (NP), patch density (PD), largest patch index (LPI), landscape division index (DIVISION), Shannon’s diversity index (SHDI) and Shannon’s evenness index (SHEI), to express the landscape pattern (Table 3).

#### 2.3.4. Correlation Analysis of Landscape Metrics with Habitat Quality and Ecological Risk

Six landscape metrics (i.e., NP, PD, LPI, DIVISION, SHDI and SHEI) were adopted, and we input the preprocessed landscape type data in 2005 and 2020 into Fragstats (version 4.2) as the data sources required to generate landscape metrics in the respective fishnet. We subsequently determined the landscape metrics distribution maps in 2005 and 2020 in ArcGIS. Lastly, the spatial analyst tools in ArcGIS were employed to generate the spatial map of the landscape metric changes in the respective fishnet from 2005 to 2020.

Based on the landscape metrics, habitat quality and ecological risk in the respective fishnet, the conversion tool in ArcGIS was adopted to convert raster data into ASCII files. We then investigated the correlations of landscape metrics with habitat quality and ecological risk by determining the Pearson correlation in IBM SPSS Statistics version 24.

Bivariate local Moran’s *I* tool in GeoDA version 1.14 was used to investigate the spatial correlation of landscape metrics with habitat quality and ecological risk in the respective fishnet. Moreover, we obtain LISA cluster maps between the landscape metrics and habitat quality as well as between the landscape metrics and ecological risk [43].

## 3. Results

### 3.1. Spatial–Temporal Change of Habitat Quality

According to the whole region and the respective distance gradient, the level of habitat quality was high in 2005 and low in 2020. Over the past 15 years (from 2005 to 2020), the habitat quality has decreased. This decline in habitat quality is reduced as the distance from the coastline increases (from gradient I to gradient IV) (Table 4).

**Figure 3:**
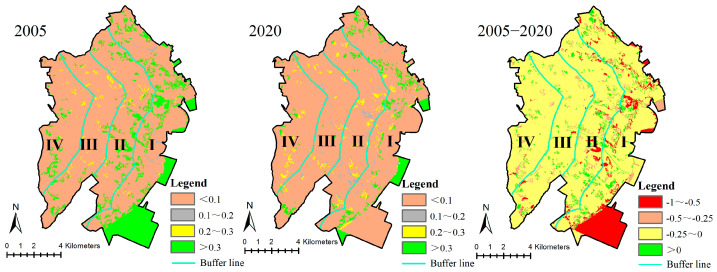
Spatial pattern of habitat quality variation. (Gradient I (0–2 km), gradient II (2–4 km), gradient III (4–6 km) and gradient IV (>6 km) from the coastline to the inland).

In 2005 and 2020, the habitat quality in most areas of the study area was low. In addition, areas with a habitat quality less than 0.1 dominated the study region, while areas with a habitat quality over 0.3 are largely concentrated in gradient I. The habitat quality in most areas of the study area exhibits a declining trend, with reductions generally between −0.25 to 0 from 2005 to 2020. The southern part of gradient I exhibits greater reductions in habitat quality (between −1 and −0.5). Only scattered areas of the town exhibit improved habitat quality (Figure 3).

### 3.2. Spatial–Temporal Changes in Ecological Risk

According to the whole region and the respective distance gradient, the ecological risk level in 2005 is less than in 2020. The increasing amounts of ecological risk in gradients I and II are significantly higher than those in gradients III and IV (Table 5).

**Figure 4:**
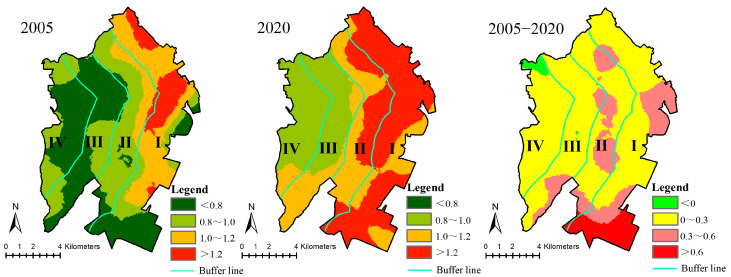
Spatial pattern of ecological risk change. (Gradient I (0–2 km), gradient II (2–4 km), gradient III (4–6 km) and gradient IV (>6 km) from the coastline to the inland).

In 2005, the high value areas (over 1.2) of ecological risk in the study area were concentrated in the central and northern parts of gradient I, while the low value areas (less than 0.8) were primarily located in gradients III and IV as well as in the southern part of gradient I. In 2020, the high value areas (over 1.2) of ecological risk were largely concentrated in areas of gradients I and II. The ecological risk values of gradients III and IV were low, ranging from 0.8 to 1.0. The ecological risk in most study areas has been increasing except for a small part of northwest between 2005 and 2020. Areas with large increases in ecological risk (over 0.6) are located in the south part of gradient I, while those with moderate increases (between 0.3 and 0.6) are concentrated in the south and east parts of gradient I and the central part of gradient II. The increase in ecological risk in most of the study area ranges from 0 to 0.3 (Figure 4).

### 3.3. Landscape Metrics Variations 

The values of other landscape metrics in the whole region in 2020 exceed those in 2005, with the exception of LPI. This indicates a higher degree of fragmentation, diversity and separation of the landscape in 2020 compared to 2005. The values of NP, PD, DIVISION, SHDI and SHEI in gradients I and II exceed those of gradients III and IV in 2005, whereas the opposite is true for 2020. The values of LPI in gradients III and IV significantly exceed those of gradients I and II in 2005, whereas the LPI value in gradients III and IV are lower than those of gradients I and II in 2020. This suggests a clear distance gradient in landscape fragmentation, diversity and separation in response to anthropogenic disturbance (Table 6).

With the exception of LPI, other landscape metrics exhibited an increase in the whole region between 2005 and 2020. The values of NP, PD, DIVISION, SHDI and SHEI have been decreasing in gradients I and II, whereas the values have been increasing in gradients III and IV. The trend of LPI in the respective gradient is in contrast to those of other landscape metrics. This indicates an increase in landscape fragmentation, separation and diversity in the study area, and the presence of a clear distance gradient characteristic of the area (Table 7).

**Figure 5:**
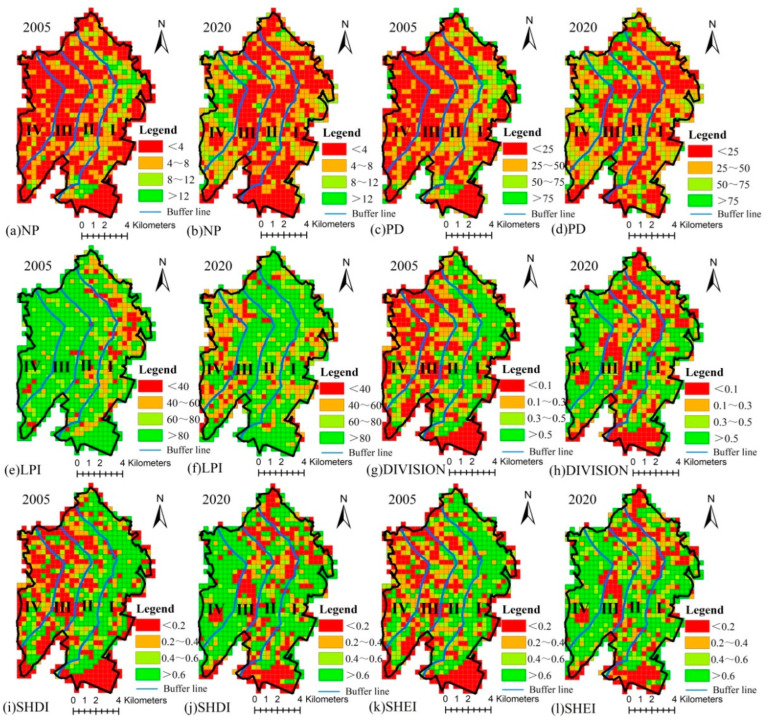
Spatial pattern of landscape metrics in 2005 and 2020. (Gradient I (0–2 km), gradient II (2–4 km), gradient III (4–6 km) and gradient IV (>6 km) from the coastline to the inland).

The high values of NP, PD, DIVISION, SHDI, and SHEI are concentrated in the gradients close to the coastline, while in 2005, the low values of the aforementioned metrics were largely located in the gradients away from the coastline. Low value areas of LPI in 2005 were located in the gradients close to the coastline, while high value areas are primarily observed in the gradients away from the coastline. The spatial pattern of landscape metrics in 2020 is inconsistent with that in 2005. This implies that the fragmentation, separation and diversity of the landscape was more prominent in 2005 and closer to the coastline than in 2020 (Figure 5).

**Figure 6:**
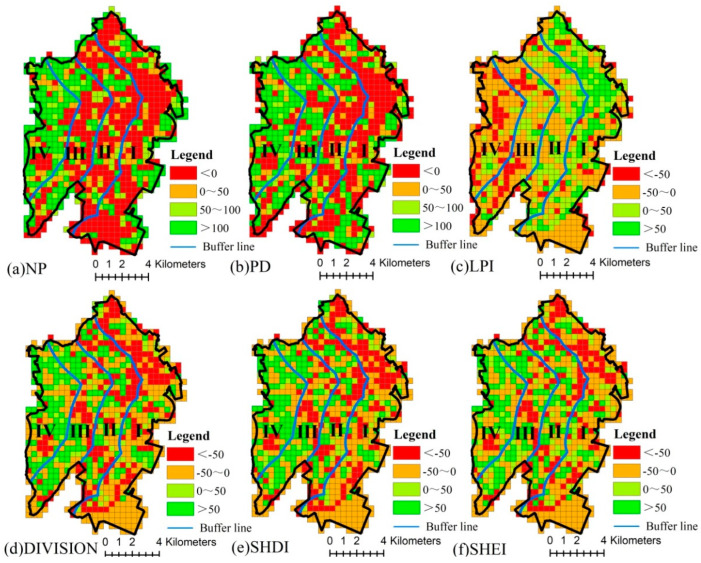
Spatial pattern of landscape metric variations from 2005 to 2020. (Gradient I (0–2 km), gradient II (2–4 km), gradient III (4–6 km) and gradient IV (>6 km) from the coastline to the inland).

The values of NP and PD generally exhibit a decreasing trend in gradients I and II from 2005 to 2020, while they are observed to increase in gradients III and IV. With the exception of gradient I, the value of LPI in most areas of the other gradients has declined. The results indicate a decrease in landscape fragmentation in the gradient region close to the coastline and an increase in the gradient regions away from the coastline. The values of DIVISION, SHDI and SHEI decrease significantly in gradient I and increase in gradient IV. This indicates that changes in landscape separation and diversity are more pronounced in areas closer to the coastline and those furthest from it (Figure 6).

### 3.4. Correlation of Habitat Quality with Landscape Metrics

We identified a positive correlation between habitat quality and NP, PD, LPI, DIVISION, SHDI and SHEI in the whole region and the respective distance gradient, while a negative correlation was observed between habitat quality and LPI in 2005 and 2020. With the exception of the data in gradient I, the correlation degree between the habitat quality and various metrics in the whole region and gradients II, III and IV in 2005 significantly exceeds that in 2020 (Table 8).

With the exception of LPI, a positive correlation was observed between the habitat quality variation and other landscape metrics variations from 2005 to 2020. The correlation degree between the habitat quality variation and landscape metrics variations in gradients I and II exceeded that in gradients III and IV (Table 9).

**Figure 7:**
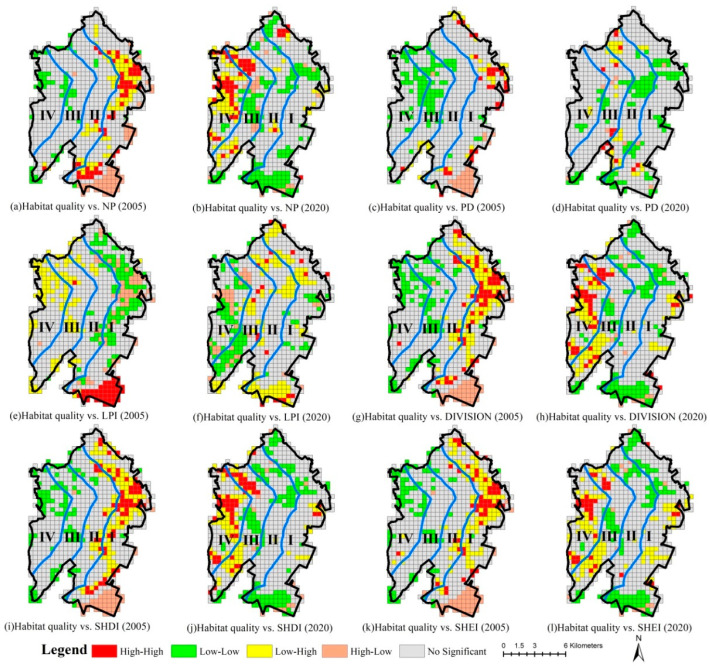
LISA cluster map between habitat quality and landscape metrics in 2005 and 2020. (Gradient I (0–2 km), gradient II (2–4 km), gradient III (4–6 km) and gradient IV (>6 km) from the coastline to the inland).

In general, the high–high, low–high and high–low correlation areas between habitat quality and NP, PD, DIVISION, SHDI and SHEI are located in gradients I and II, while low–low correlation areas are primarily observed in gradients III and IV in 2005. Overall, the high–high, low–low and high–low correlation areas between habitat quality and LPI are located in gradient I, while the low–high correlation areas are mostly found in gradients III and IV in 2005. The spatial distribution of the respective correlation type area in the LISA cluster map in 2020 is inconsistent with that in 2005 (Figure 7).

**Figure 8:**
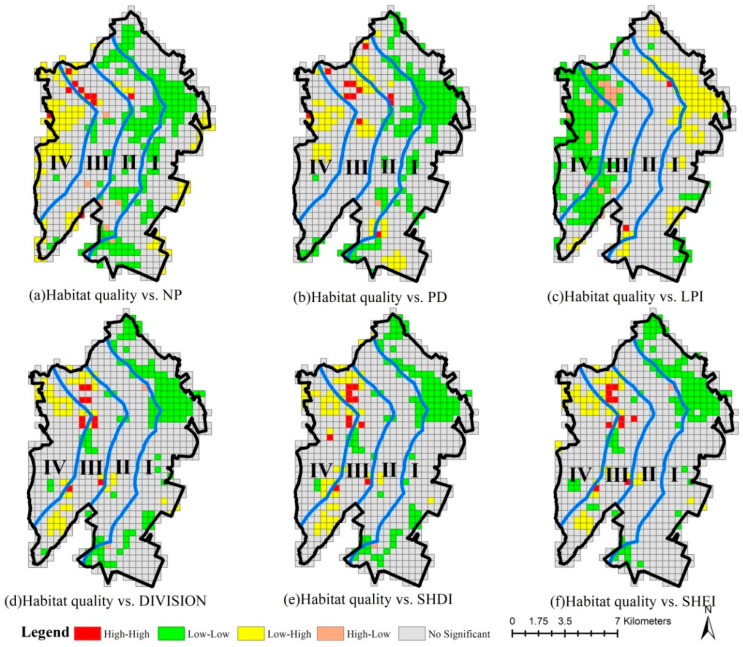
LISA cluster map between habitat quality variation and landscape metric changes from 2005 to 2020. (Gradient I (0–2 km), gradient II (2–4 km), gradient III (4–6 km) and gradient IV (>6 km) from the coastline to the inland).

The LISA cluster maps are dominated by low–low and low–high correlation areas between habitat quality variation and different landscape metrics variations from 2005 to 2020. On the whole, the low–low correlation areas between habitat quality variation and NP, PD, DIVISION, SHDI and SHEI variations are located in gradients I and I over the past 15 years, while the low–high correlation areas are observed primarily in gradients III and IV. However, the low–low correlation areas between habitat quality variation and LPI change are generally located in gradients III and IV, and the low–high correlation areas are largely found in gradients I and II (Figure 8).

### 3.5. Correlation of Ecological Risk with Landscape Metrics

We observed a positive correlation between ecological risk and NP, PD, DIVISION, SHDI and SHEI in the whole region and the respective gradient, as well as a negative correlation between ecological risk and LPI in 2005 and 2020. The degree of correlation between ecological risk and multiple landscape metrics in gradients I and II in 2005 exceeded that in 2020, while the degree of correlation between ecological risk and multiple landscape metrics in gradients III and IV in 2005 was less than that in 2020 (Table 10).

With the exception of the landscape metric LPI, a positive correlation was observed between other landscape metrics variations and the ecological risk changes in the whole region and the respective gradient from 2005 to 2020. The correlation degree between LPI change and ecological risk change exceeded (insignificantly) that between other landscape metrics variations and ecological risk change (Table 11).

**Figure 9:**
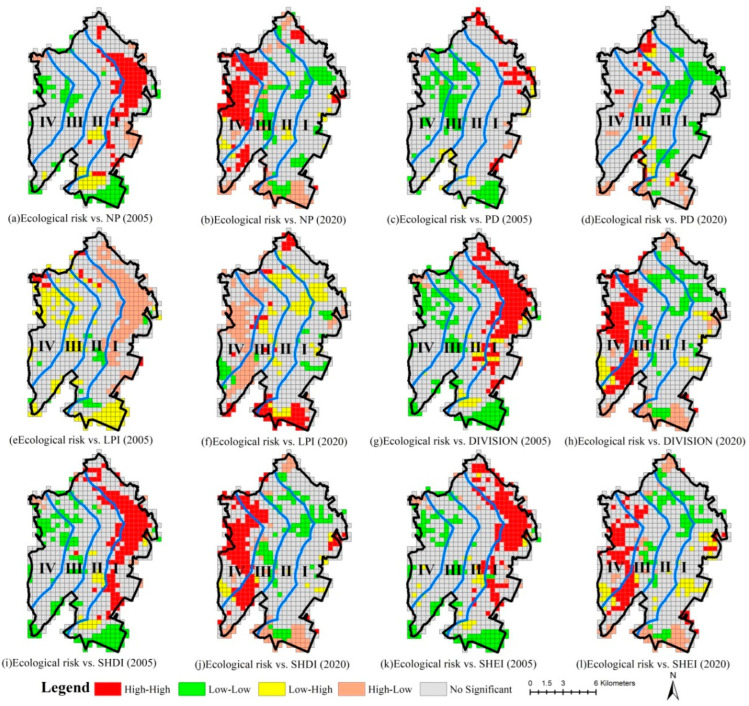
LISA cluster map between ecological risk and landscape metrics in 2005 and 2020. (Gradient I (0–2 km), gradient II (2–4 km), gradient III (4–6 km) and gradient IV (>6 km) from the coastline to the inland).

In general, the high–high correlation areas between ecological risk and NP, PD, DIVISION, SHDI and SHEI in 2005 were located in gradient I, while the low–low correlation areas were found in gradients III and IV. Moreover, the high–low correlation areas between ecological risk and LPI were largely located in gradient I, and the low–high correlation areas were concentrated in gradients I, III and IV. In contrast, the high–high correlation areas between ecological risk and NP, PD, DIVISION, SHDI and SHEI in 2020 were concentrated in gradients III and IV, while the low–low correlation areas were mainly located in gradients I and II. On the whole, the high–low correlation areas between ecological risk and LPI largely belong to gradients III and IV, while the low–high correlation areas were concentrated in gradients I and II in 2020 (Figure 9). 

**Figure 10:**
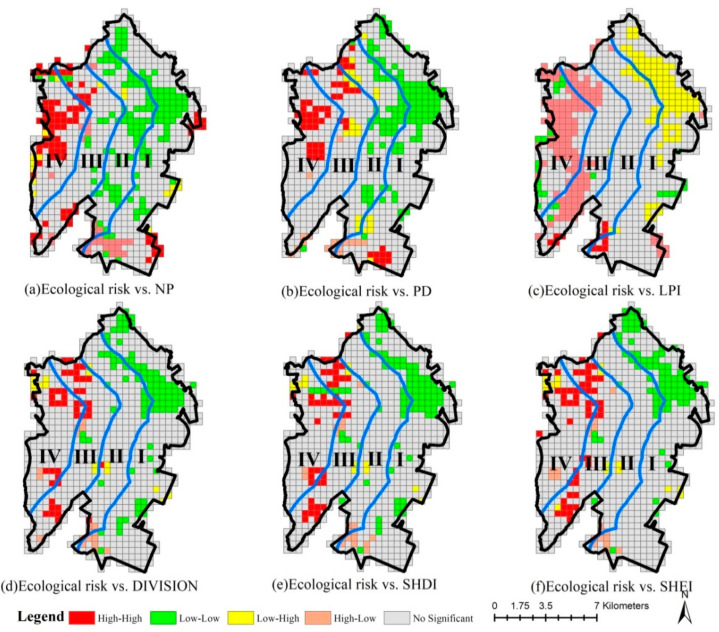
LISA cluster map between ecological risk change and landscape metrics changes from 2005 to 2020. (Gradient I (0–2 km), gradient II (2–4 km), gradient III (4–6 km) and gradient IV (>6 km) from the coastline to the inland).

The low–low correlation areas between ecological risk change and NP, PD, DIVISION, SHDI and SHEI variations were concentrated in gradients I and II from 2005 to 2020, while the high–high correlation areas were located mainly in gradients III and IV. The low–high correlation areas between ecological risk change and LPI change are concentrated in gradient I, while the high–low correlation areas are generally found in gradients III and IV (Figure 10).

## 4. Discussion

Although quantitative studies of the impact of human activities on the ecological environment have been reported, the mechanisms driving changes in habitat quality and ecological risk from a landscape perspective and their correlations with landscape indicators have not been clarified. Investigating these issues is key to clarifying the impact mechanisms of landscape change on the ecological environment under anthropogenic disturbance. The study of habitat quality and ecological risk in coastal areas, from a landscape perspective, can provide clear information for landscape optimization, regional landscape planning and differentiated management strategies for different landscape types.

### 4.1. Spatial Pattern and Variations of Habitat Quality and Ecological Risk

**Figure 11:**
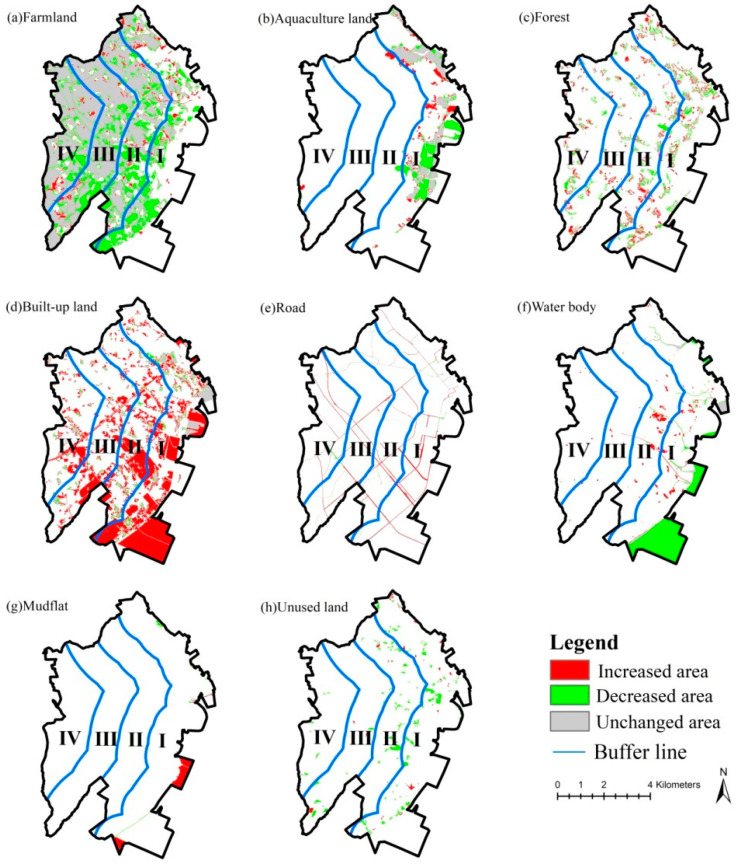
Spatial pattern of landscape type variations in the study area from 2005 to 2020. (Gradient I (0–2 km), gradient II (2–4 km), gradient III (4–6 km) and gradient IV (>6 km) from the coastline to the inland).

The results reveal that the gradient characteristics of habitat quality and ecological risk in coastal regions are correlated with the spatial pattern of different landscape types of different gradients. The gradients close to the coastline refer to the key area of human development, and the proportions of human-dominated landscapes (e.g., built-up land) are high, thereby threatening the habitat and causing a high ecological risk. On the other hand, the gradients far from the coastline are dominated by farmland for agricultural production and insignificantly threaten the habitat, thereby causing a low ecological risk (Table 12). Moreover, the habitat quality in coastal regions decreases, and the ecological risk increases under the robust human disturbance, which is consistent with the results of Li [21] and Zhang [4]. In this paper, the decline of habitat quality and the increase in ecological risk in coastal regions are correlated with the rapid expansion of built-up land and the significant reduction of farmland and water bodies (Table 13), which is consistent with the results of Zhang [4] and Tang [44]. In addition, habitat quality degradation and ecological risk enhancement are significant in the gradients close to the coastline, which is correlated with higher development intensity in this gradient than in the gradients far from the coastline. In other words, the farmland and the water bodies have obviously declined more in near-coastline gradients as the built-up land has expanded (Table 13 and Figure 11). 

### 4.2. Correlation of Habitat Quality and Ecological Risk with Landscape Metrics

In this paper, the Pearson correlation and spatial autocorrelation are adopted to examine the correlation of landscape metrics with habitat quality and ecological risk. As revealed from the results, habitat quality and ecological risk were correlated with landscape metrics, thereby agreeing with the conclusions drawn by Zhu [45] and Yan [46]. However, the correlations of different landscape metrics with habitat quality and ecological risk are different, and the reason for this result is that different landscape metrics respond differently to human activities. Rapid urbanization has changed the original human activity types dominated by agricultural production. In particular, the diversity of landscape types and the number of patches has increased, and the maximal landscape (agricultural landscape) area has decreased. As a consequence, landscape fragmentation is promoted and the degree of landscape separation increases, thereby reducing the habitat quality and increasing the ecological risk (Table 4, Table 5 and Table 7). Thus, NP, PD, DIVISION, SHDI and SHEI are positively correlated with habitat quality and ecological risk, while LPI is negatively correlated with habitat quality and ecological risk (Table 9 and Table 11). Note that spatial heterogeneity is observed from the correlation of the landscape metrics with habitat quality and ecological risk for the different distance gradients from the coastline. For example, from 2005 to 2020, the landscape metrics decreased, except for LPI in gradients I and II, while they increased in gradients III and IV (Table 7). Moreover, the ecological risk in gradients I and II rapidly declined, while slower reductions were observed in gradients III and IV (Table 5). As a consequence, a low–low spatial correlation area was defined in gradients I and II between the landscape metrics and ecological risk from 2005 to 2020, and a high–high spatial correlation area was defined in gradients III and IV (Figure 10).

### 4.3. Ecological Management and Landscape Planning

Following the rapid urbanization, economic development and population explosion of coastal regions, human activities have imposed more pressure on the ecological environment. This has caused serious habitat degradation and intensified ecological risks. In the future, the green development model should be implemented continuously, and the ecological protection red line should be delimited in accordance with the social and economic development trends and the distance from the coast. In addition, ecological management zones should be established, differentiated regional management and control measures should be adopted, and the core ecological function area should be primarily protected. The ecological environment quality in the coastal region could also be improved by landscape planning. To coordinate the landscape planning with the ecological environment in coastal regions, specific measures can be taken, including broadening the area of natural landscape (forest, water body and mudflat), protecting the landscape types with high ecological functions, regulating the speed of built-up land expansion and the development of water bodies, increasing the level of economical and intensive use of built-up land, and facilitating the protection of farmland and aquaculture land.

### 4.4. Limitations

As the assessment results of the habitat quality module in the InVEST model are obtained based on expert experience scoring, this leads to greater human interference and uncertainty in the assessment results. Therefore, in the future, the influence of human factors in the parameter setting should be reduced to improve the accuracy of the assessment results [16,19]. As the rate of socioeconomic development in China’s coastal areas slows [47], the impact of human activities on the ecological environment is bound to change in the future. This study has yet to examine the changing status of habitat quality and landscape ecological risk under the influence of complex human activities in the future, a key direction for future research.

## 5. Conclusions

The significant conclusions of this study are as follows: (1) Coastal regions of China exhibit declining habitat quality and deteriorating ecological risks. This is linked to rapid socioeconomic development resulting in the increasing intensity of human activity. Changes in habitat quality and ecological risk are accentuated by increasing human activity in areas closer to the coastline. This is due to the significant expansion of built-up land and the serious destruction of nature in near-coastline gradients. (2) The fragmentation, diversity and separation of China’s coastal regional landscapes have increased under the influence of intense human activity. This leads the landscape metrics to positively correlate with changes in ecological risk and habitat quality. (3) Differentiated regional ecological management and control measures should be applied to different distance gradient zones along the coast. Moreover, the expansion of landscape types that characterize high intensity human activity (e.g., built-up land and roads) should be constrained. At the same time, the protection of natural landscapes (e.g., forests and bodies of water) can be enhanced and ecological protection red lines should be designated.

## Figures and Tables

**Table 1:** Maximal impact distance and weight of the threat factors.

Factor	Farmland	Aquaculture Land	Built-Up Land	Road
Maximal Impact Distance/km	1	0.5	2	0.5
Weight	0.4	0.3	0.6	0.3

**Table 2:** Sensitivity of the threat factors to various habitat types.

Habitat Type	Farmland	Aquaculture Land	Built-Up Land	Road
Forest	0.3	0.1	0.5	0.3
Water Body	0.4	0.6	0.8	0.2
Mudflat	0.1	0.7	0.4	0.1
Unused Land	0.2	0.1	0.3	0.1

**Table 3:** Calculation method and ecological significance of selected landscape metrics.

Metrics	Formula	Ecological Significance
NP	NP=n, *n* is the number of patches.	Higher value indicates more severe landscape fragmentation.
PD	PD=n/A, *A* is the total landscape area (m^2^).	Reflects the degree of landscape segmentation, which together with NP, denotes the degree of landscape fragmentation.
LPI	LPI=Maxi=1n(ai)A×100, *a_i_* is the area of patch *i* (m^2^).	Determines landscape dominance and reflects the direction and strength of human activities.
DIVISION	DIVISION=1−∑j=1naijA, *a_ij_* is the area of patch *i* of landscape type *j* (m^2^).	Indicates the degree of landscape separation.
SHDI	$\mathrm{SHDI}=-\sum_{i=1}^{m}\left[P_{i}\ln(P_{i})\right]$, *P_i_* is the proportion of the landscape occupied by patch type *i*; *and m* is the number of patch types present in the landscape.	Reflects landscape heterogeneity and represents the degree of landscape diversity.
SHEI	SHEI=−∑i=1mPilog(Pi)logm, *P_i_* is the proportion of landscape occupied by patch type *i*; and is the number of patch types present in the landscape.	Ecological significance of SHEI is consistent with that of SHDI.

**Table 4:** Variations of habitat quality in the study area.

Area	2005	2020	Variation from 2005 to 2020
Gradient I	0.2621	0.0736	−0.1885
Gradient II	0.0542	0.0238	−0.0304
Gradient III	0.0249	0.0157	−0.0092
Gradient IV	0.0228	0.0158	−0.0070
Whole Region	0.1237	0.0402	−0.0835

**Table 5:** Change of ecological risk in study area.

Area	2005	2020	Variation from 2005 to 2020
Gradient I	0.99	1.17	0.18
Gradient II	0.92	1.04	0.12
Gradient III	0.77	0.81	0.04
Gradient IV	0.76	0.77	0.01
Whole Region	0.89	0.99	0.10

**Table 6:** Values of landscape metrics in 2005 and 2020.

Metric	Gradient I		Gradient II		Gradient III		Gradient IV		Whole Region	
	2005	2020	2005	2020	2005	2020	2005	2020	2005	2020
NP	5.12	4.71	5.03	4.34	3.47	5.63	3.15	7.91	4.36	5.47
PD	42.62	34.98	32.87	29.53	25.70	44.43	22.34	45.24	33.95	41.10
LPI	74.41	77.75	79.53	81.88	88.53	71.66	89.69	62.97	81.58	74.14
DIVISION	0.34	0.30	0.29	0.26	0.17	0.38	0.16	0.49	0.26	0.35
SHDI	0.57	0.49	0.49	0.43	0.29	0.62	0.27	0.82	0.44	0.58
SHEI	0.45	0.43	0.42	0.39	0.27	0.49	0.27	0.60	0.37	0.47

**Table 7:** Change rate of landscape metrics from 2005 to 2020.

Metric	Gradient I	Gradient II	Gradient III	Gradient IV	Whole Region
NP	−8.01%	−13.72%	62.25%	151.11%	25.46%
PD	−17.93%	−10.16%	72.88%	102.51%	21.06%
LPI	4.49%	2.95%	−19.06%	−29.79%	−9.12%
DIVISION	−11.76%	−10.34%	123.53%	206.25%	34.62%
SHDI	−14.04%	−12.24%	113.79%	203.70%	31.82%
SHEI	−4.44%	−7.14%	81.48%	122.22%	27.03%

**Table 8:** Correlation coefficients between habitat quality and landscape metrics in 2005 and 2020.

Metric	Gradient I		Gradient II		Gradient III		Gradient IV		Whole Region	
	2005	2020	2005	2020	2005	2020	2005	2020	2005	2020
NP	0.089	0.598 **	0.637 **	0.413 **	0.731 **	0.415 **	0.638 **	0.517 **	0.383 **	0.548 **
PD	0.083	0.494 **	0.524 **	0.430 **	0.640 **	0.368 **	0.579 **	0.540 **	0.406 **	0.510 **
LPI	−0.013	−0.590 **	−0.814 **	−0.437 **	−0.727 **	−0.448 **	−0.660 **	−0.527 **	−0.459 **	−0.565 **
DIVISION	0.011	0.611 **	0.818 **	0.446 **	0.774 **	0.462 **	0.662 **	0.550 **	0.461 **	0.582 **
SHDI	0.001	0.656 **	0.814 **	0.484 **	0.779 **	0.481 **	0.702 **	0.592 **	0.468 **	0.618 **
SHEI	0.023	0.524 **	0.799 **	0.435 **	0.761 **	0.458 **	0.657 **	0.503 **	0.446 **	0.525 **

Note: ** indicates significant correlation at the significance level of 0.01.

**Table 9:** Correlation coefficients between habitat quality variation and landscape metrics variations from 2005 to 2020.

Metric	Gradient I	Gradient II	Gradient III	Gradient IV	Whole Region
NP	0.458 **	0.421 **	0.305 **	0.309 **	0.472 **
PD	0.421 **	0.442 **	0.270 **	0.333 **	0.431 **
LPI	−0.395 **	−0.446 **	−0.268 **	−0.309 **	−0.516 **
DIVISION	0.405 **	0.429 **	0.253 **	0.390 **	0.467 **
SHDI	0.459 **	0.443 **	0.262 **	0.308 **	0.489 **
SHEI	0.431 **	0.413 **	0.245 **	0.320 **	0.436 **

Note: ** indicates significant correlation at the significance level of 0.01.

**Table 10:** Correlation coefficients between ecological risk and landscape metrics in 2005 and 2020.

Metric	Gradient I		Gradient II		Gradient III		Gradient IV		Whole Region	
	2005	2020	2005	2020	2005	2020	2005	2020	2005	2020
NP	0.432 **	0.015	0.195 **	0.098	0.059	0.287 **	0.036	0.299 **	0.365 **	0.202 **
PD	0.359 **	0.140 **	0.157	0.143 *	0.021	0.139 *	0.143	0.308 **	0.319 **	0.244 **
LPI	−0.475 **	−0.005	−0.250 **	−0.049	−0.097	−0.296 **	−0.023	−0.185*	−0.436 **	−0.213 **
DIVISION	0.474 **	0.015	0.250 **	0.050	0.095	0.307 **	0.029	0.213 **	0.433 **	0.226 **
SHDI	0.464 **	0.035	0.241 **	0.039	0.102	0.357 **	0.015	0.292 **	0.435 **	0.258 **
SHEI	0.436 **	0.023	0.235 **	0.060	0.095	0.239 **	0.035	0.137	0.419 **	0.156 **

* Indicates significant correlation at the significance level of 0.05; ** indicates significant correlation at the significance level of 0.01.

**Table 11:** Correlation coefficients between ecological risk change and landscape metrics variations from 2005 to 2020.

Metric	Gradient I	Gradient II	Gradient III	Gradient IV	Whole Region
NP	0.295 **	0.109	0.243 **	0.094	0.322 **
PD	0.276 **	0.162 *	0.033	0.207 **	0.299 **
LPI	−0.338 **	−0.206 **	−0.309 **	−0.266 *	−0.383 **
DIVISION	0.250 **	0.114	0.178 **	0.100	0.267 **
SHDI	0.258 **	0.103	0.188 **	0.113	0.276 **
SHEI	0.175 **	0.082	0.166 *	0.075	0.219 **

* Indicates significant correlation at the significance level of 0.05; ** indicates significant correlation at the significance level of 0.01.

**Table 12:** Area of various landscape types in 2005 and 2020 (ha).

Landscape Types	Gradient I		Gradient II		Gradient III		Gradient IV		Whole Region	
	2005	2020	2005	2020	2005	2020	2005	2020	2005	2020
Farmland	1671	960	2294	1637	2510	2068	2029	1778	8504	6444
Aquaculture Land	1152	910	27	32	3	4	4	7	1187	953
Forest	379	367	249	238	137	138	113	111	879	853
Built-Up Land	545	2251	178	821	106	524	85	316	914	3915
Road	24	135	17	66	23	59	23	49	87	310
Water Body	1082	211	12	70	1	12	3	10	1102	304
Mudflat	47	210	0	0	0	0	0	0	48	212
Unused Land	187	42	97	10	38	13	14	0	335	65

**Table 13:** Variations of each landscape type area from 2005 to 2020 (ha).

Area	Farmland	Aquaculture Land	Forest	Built-Up Land	Road	Water Body	Mudflat	Unused Land
Gradient I	−711	−242	−12	1706	111	−871	163	−145
Gradient II	−657	5	−11	643	49	58	0	−87
Gradient III	−442	1	1	418	36	11	0	−25
Gradient IV	−251	3	−2	231	26	7	0	−14
Whole Region	−2060	−234	−26	3001	223	−798	164	−271

## Data Availability

The data used to support the findings of this study are available from the corresponding author upon request.

## References

[1] Zhang H.B., Liu Y.Q., Xu Y., Han S., Wang J. (2020). Impacts of Spartina alterniflora expansion on landscape pattern and habitat quality: A case study in Yancheng coastal wetland, China. Appl. Ecol. Environ. Res..

[2] Tian P., Cao L.D., Li J.L., Pu R.L., Gong H.B., Li C.D. (2021). Landscape characteristics and ecological risk assessment based on multi-scenario simulations: A case study of Yancheng coastal wetland, China. Sustainability.

[3] Zhang W., Chang W.J., Zhu Z.C., Hui Z. (2020). Landscape ecological risk assessment of Chinese coastal cities based on land use change. Appl. Geogr..

[4] Zhang X.R., Song W., Lang Y.Q., Feng X.M., Yuan Q.Z., Wang J.T. (2020). Land use changes in the coastal zone of China’s Hebei Province and the corresponding impacts on habitat quality. Land Use Policy.

[5] Krebs J.M., Bell S.S., McIvor C.C. (2014). Assessing the link between coastal urbanization and the quality of nekton habitat in mangrove tidal tributaries. Estuar. Coast..

[6] Paterson G.B., Smart G., McKenzie P., Cook S. (2019). Prioritising sites for pollinators in a fragmented coastal nectar habitat network in Western Europe. Landsc. Ecol..

[7] Zhai T.L., Wang J., Fang Y., Qin Y., Huang L.Y., Chen Y. (2020). Assessing ecological risks caused by human activities in rapid urbanization coastal areas: Towards an integrated approach to determining key areas of terrestrial-oceanic ecosystems preservation and restoration. Sci. Total. Environ..

[8] Yu W.W., Zhang L.P., Ricci P.F., Chen B., Huang H. (2015). Coastal ecological risk assessment in regional scale: Application of the relative risk model to Xiamen Bay, China. Ocean Coast. Manag..

[9] Guan L.S., Chen Y., Wilson J.A. (2017). Evaluating spatio-temporal variability in the habitat quality of Atlantic cod (*Gadus morhua*) in the Gulf of Maine. Fish. Oceanogr..

[10] Abreu F.E.L., Martins S.E., Fillmann G. (2021). Ecological risk assessment of booster biocides in sediments of the Brazilian coastal areas. Chemosphere.

[11] Ding Q.L., Chen Y., Bu L.T., Ye Y.M. (2021). Multi-scenario analysis of habitat quality in the Yellow River delta by coupling FLUS with InVEST model. Int. J. Environ. Res. Public Health.

[12] Meng L., Cicchetti G., Chintala M. (2004). Nekton habitat quality at shallow water sites in two Rhode island coastal systems. Estuaries.

[13] Landry J.B., Golden R.R. (2019). In situ effects of shoreline type and watershed land use on submerged aquatic vegetation habitat quality in the Chesapeake and Mid-Atlantic coastal bays. Estuar. Coast..

[14] Yeung C., Yang M.S. (2018). Spatial variation in habitat quality for juvenile flatfish in the southeastern Bering Sea and its implications for productivity in a warming ecosystem. J. Sea. Res..

[15] Luo X.X., Lin S., Yang J.Q., Shen J.Y., Fan Y.Q., Zhang L.J. (2017). Benthic habitat quality assessment based on biological indices in Xiaoqing River estuary and its adjacent sea of Laizhou Bay, China. J. Ocean. U Chin..

[16] Zhang X., Liao L.Y., Xu Z.D., Zhang J.Y., Chi M.W., Lan S.R., Gan Q.C. (2022). Interactive effects on habitat quality using InVEST and GeoDetector models in Wenzhou, China. Land.

[17] Wang B.X., Cheng W.M. (2022). Effects of land use/cover on regional habitat quality under different geomorphic types based on InVEST model. Remote Sens..

[18] Wei Q.Q., Abudureheman M., Halike A., Yao K.X., Yao L., Tang H., Tuheti B. (2022). Temporal and spatial variation analysis of habitat quality on the PLUS-InVEST model for Ebinur Lake Basin, China. Ecol. Indic..

[19] Li Y.N., Duo L.H., Zhang M., Yang J.Y., Guo X.F. (2022). Habitat quality assessment of mining cities based on InVEST model-a case study of Yanshan County, Jiangxi Province. Int. J. Coal. Sci. Technol..

[20] Landis W.G. (2004). Ecological risk assessment conceptual model formulation for nonindigenous species. Risk. Anal..

[21] Li J.L., Pu R.L., Gong H.B., Luo X., Ye M.Y., Feng B.X. (2017). Evolution characteristics of landscape ecological risk patterns in coastal zones in Zhejiang Province, China. Sustainability.

[22] Liu Y.C., Liu Y.X., Li J.L., Lu W.Y., Wei X.L., Sun C. (2018). Evolution of landscape ecological risk at the optimal scale: A case study of the open coastal wetlands in Jiangsu, China. Int. J. Environ. Res. Public Health.

[23] Hua L.Z., Liao J.F., Chen H.X., Chen D.K., Shao G.F. (2018). Assessment of ecological risks induced by land use and land cover changes in Xiamen City, China. Int. J. Sust. Dev. World. Ecol..

[24] Li R.X., Yuan Y., Li C.W., Sun W., Yang M., Wang X.R. (2020). Environmental health and ecological risk assessment of soil heavy metal pollution in the coastal cities of estuarine bay-A case study of Hangzhou bay, China. Toxics.

[25] Smith J.A.M., Niles L.J., Hafner S., Modjeski A., Dillingham T. (2020). Beach restoration improves habitat quality for American horseshoe crabs and shorebirds in the Delaware Bay, USA. Mar. Ecol. Prog. Ser..

[26] Soniat T.M., Conzelmann C.P., Byrd J.D., Roszell D.P., Bridevaux J.L., Suir K.J., Colley S.B. (2013). Predicting the effects of proposed Mississippi river diversions on oyster habitat quality; application of an oyster habitat suitability index model. J. Shellfish. Res..

[27] Omar H., Cabral P. (2020). Ecological risk assessment based on land cover changes: A case of Zanzibar (Tanzania). Remote Sens..

[28] Li X.Q., Lu Y.L., Shi Y.J., Wang P., Cao X.H., Cui H.T., Zhang M., Du D. (2022). Effects of urbanization on the distribution of polycyclic aromatic hydrocarbons in China’s estuarine rivers. Environ. Pollut..

[29] Sajjad M., Chan J.C.L., Kanwal S. (2020). Integrating spatial statistics tools for coastal risk management: A case-study of typhoon risk in mainland China. Ocean Coast. Manag..

[30] Meng Z.Q., Long L.B., She Q.N., Cheng D.Y., Liu M. (2018). Assessment of ecological conditions over China’s coastal areas based on land use / cover change. Chin. J. Appl. Ecol..

[31] Aneseyee A.B., Noszczyk T., Soromessa T., Elias E. (2020). The InVEST habitat quality model associated with land use/cover changes: A qualitative case study of the Winike watershed in the Omo-Gibe basin, southwest Ethiopia. Remote Sens..

[32] Yohannes H., Soromessa T., Argaw M., Dewan A. (2021). Spatio-temporal changes in habitat quality and linkage with landscape characteristics in the Beressa watershed, Blue Nile basin of Ethiopian highlands. J. Environ. Manag..

[33] Sharp R., Douglass J., Wolny S., Arkema K., Bernhardt J., Bierbower W., Chaumont N., Denu D., Fisher D., Glowinski K. (2022). InVEST 3.12.1.post6+ug.g3c8454b User’s Guide. https://storage.googleapis.com/releases.naturalcapitalproject.org/invest-userguide/latest/index.html.

[34] Han H.Q., Zhang Y.J., Liu Y., Yu X., Wang J.W. (2022). Spatiotemporal changes of the habitat quality and the human activity intensity and their correlation in mountainous cities. J. Environ. Eng. Landsc..

[35] Xu L.T., Chen S.S., Xu Y., Li G.Y., Su W.Z. (2019). Impacts of land-use change on habitat quality during 1985-2015 in the Taihu lake basin. Sustainability.

[36] Wang B.B., Ding M.J., Li S.C., Liu L.S., Ai J.H. (2020). Assessment of landscape ecological risk for a cross-border basin: A case study of the Koshi River Basin, central Himalayas. Ecol. Indic..

[37] Xie H.L., Wen J.M., Chen Q.R., Wu Q. (2021). Evaluating the landscape ecological risk based on GIS: A case-study in the Poyang lake region of China. Land Degrad. Dev..

[38] Gao H., Song W. (2022). Assessing the landscape ecological risks of land-use change. Int. J. Environ. Res. Public Health.

[39] Chen J., Dong B., Li H.R., Zhang S.S., Peng L., Fang L., Zhang C.B., Li S. (2020). Study on landscape ecological risk assessment of Hooded Crane breeding and overwintering habitat. Environ. Res..

[40] Zhao Y.Y., Kasimu A., Liang H.W., Reheman R. (2022). Construction and restoration of landscape ecological network in Urumqi city based on landscape ecological risk assessment. Sustainability.

[41] Gao B.P., Wu Y.M., Li C., Zheng K.J., Wu Y., Wang M.J., Fan X., Ou S.Y. (2022). Multi-scenario prediction of landscape ecological risk in the Sichuan-Yunnan ecological barrier based on terrain gradients. Land.

[42] Mayer A.L., Buma B., Davis A., Gagné S.A., Loudermilk E.L., Scheller R.M., Schmiegelow F.K.A., Wiersma Y.F., Franklin J. (2016). How landscape ecology informs global land-change science and policy. BioScience.

[43] Yao K.X., Halike A., Chen L.M., Wei Q.Q. (2022). Spatiotemporal changes of eco-environmental quality based on remote sensing-based ecological index in the Hotan Oasis, Xinjiang. J. Arid. Land.

[44] Tang F., Fu M.C., Wang L., Song W.J., Yu J.F., Wu Y.B. (2021). Dynamic evolution and scenario simulation of habitat quality under the impact of land-use change in the Huaihe River Economic Belt, China. PLoS ONE.

[45] Zhu C.M., Zhang X.L., Zhou M.M., He S., Gan M.Y., Yang L.X., Wang K. (2020). Impacts of urbanization and landscape pattern on habitat quality using OLS and GWR models in Hangzhou, China. Ecol. Indic..

[46] Yan J.X., Li G., Qi G.P., Qiao H.Q., Nie Z.G., Huang C.X., Kang Y.X., Sun D.Y., Zhang M.H., Kang X. (2021). Landscape ecological risk assessment of farming-pastoral ecotone in China based on terrain gradients. Hum. Ecol. Risk. Assess..

[47] Wu S.Q., Yang D.G., Xia F.Q., Zhang X.H., Huo J.W., Cai T.Y., Sun J. (2022). The Effect of Labor Reallocation and Economic Growth in China. Sustainability.

